# Use of the Instagram Hashtags #winemom and #momjuice Among Mothers During the COVID-19 Pandemic: Descriptive, Cross-sectional Study

**DOI:** 10.2196/28991

**Published:** 2021-05-18

**Authors:** Corey H Basch, Zoe C Meleo-Erwin, Jan Mohlman, Joseph Fera, Nasia Quinones

**Affiliations:** 1 Department of Public Health William Paterson University Wayne, NJ United States; 2 Department of Psychology William Paterson University Wayne, NJ United States; 3 Department of Mathematics Lehman College Bronx, NY United States

**Keywords:** Instagram, alcohol consumption, COVID-19, social media, communication, parenting

## Abstract

**Background:**

The tendency of parents to consume alcohol during the COVID-19 pandemic is likely to be moderated by pandemic-related stress combined with the ongoing demands of childcare and home-based education, which are reported to be more burdensome for females than males.

**Objective:**

The purpose of this study was to describe alcohol-related content posted by mothers on Instagram during the COVID-19 pandemic.

**Methods:**

Using two popular hashtags, #momjuice and #winemom, 50 Instagram posts on each were collected from the “top posts” tab. The coding categories were created inductively and were as follows: displays alcohol (drinking/holding alcohol or alcohol itself), person is making alcoholic beverages, type of alcohol featured or discussed, highlights anxiety and/or depression/mental state, highlights struggling (in general), highlights parenting challenges, encourages alcohol consumption, discourages alcohol consumption, features a person wearing clothing or shows products promoting alcohol, promotes alcohol rehabilitation, highlights caffeine to alcohol daily transition throughout the day, and highlights other drugs besides caffeine and alcohol.

**Results:**

Overall, the 100 selected posts had a total of 5108 comments and 94,671 likes. The respective averages were 51.08 (SD 77.94) and 946.71 (SD 1731.72). A majority (>50%) of the posts reviewed encouraged alcohol consumption (n=66) and/or displayed alcohol (n=56). Of the 66 that encouraged and/or displayed alcohol, the common type of alcohol discussed or featured was wine (n=55). Only 6 posts discouraged alcohol use and only 4 provided the audience with a disclaimer. None of the videos promoted or endorsed alcohol rehabilitation in any way. Only 37 posts highlighted struggle. However, these posts garnered more than a majority of the likes (n=50,034, 52.3%). Posts that showed struggle received an average of 1359.57 (SD 2108.02) likes. Those that did not show struggle had an average of 704.24 (SD 1447.46) likes. An independent one-tailed *t* test demonstrated this difference to be statistically significant (*P*=.0499).

**Conclusions:**

The findings of this investigation suggest that though these hashtags ostensibly exist to valorize excess alcohol consumption, they may be serving as a support system for mothers who are experiencing increased burdens and role stress during the pandemic. Given the strains placed on mothers overall and especially during the COVID-19 pandemic, efforts must be taken to increase access to and affordability of telehealth-based mental health care.

## Introduction

Much media attention has been paid to the burdens that the COVID-19 pandemic has placed upon women in general and mothers specifically. Though previous studies have noted that representations of drinking are commonplace on Instagram, these studies tend to be focused on youth. Given that recent research suggests an alarming increase in alcohol consumption among women during the pandemic, an investigation into how this population represents alcohol use on social media is warranted. This study sought to describe and analyze posts focused on drinking among mothers on Instagram on several content dimensions (eg, promoting alcohol consumption, stress or struggle, social support), which may clarify the attitudes and motivating factors of an online subgroup of drinking mothers.

Neither the mental health nor the economic effects of the pandemic in the United States has been borne evenly. Regarding the economic fallout, alarm bells were rung regarding the potential for a COVID-19 “she-cession” given that women constituted the majority of those who either lost employment in spring of 2020 or took a leave of absence from their positions in order to care for children learning from home [1]. A recent Census Bureau report [2] describes the labor market losses women have faced over the past year as “devastating.” The authors note that as of mid-January of this year, approximately 10 million women in the United States living with school-age children were not actively in the labor market, an increase of approximately 1.4 million since January 2019 [2]. Though mothers were hit harder by the economic effects of the pandemic compared to fathers, the gap in the work status between the two groups has narrowed substantially over the past several months [2].

Nevertheless, areas of considerable concern remain. First, as the US Bureau of Labor Statistics has documented, women continue to carry far more of the burden for domestic and childcare labor than do men [3,4]. When mothers return to the labor market, they must once again balance domestic labor with paid labor [2]. This is complicated by the fact that across the country, many children continue to learn from home—whether because school districts are still operating in a remote format or because parents have chosen this mode of delivery out of an abundance of caution. This balancing act is made all the more complicated by pandemic-specific “care economy” work [5] undertaken by women wherein women are attending to the emotional well-being of family members. Second, previous studies have documented that a temporary departure from the labor force (eg, for childbirth) may have long-term negative effects on women’s earning power [2]. Given this, pandemic-related labor-force participation gaps may suppress the economic position of women for years to come.

While disparities based on race and ethnicity are not a focus of this paper, it is important to note that among women, the labor market effects of the pandemic have been uneven, with women of color facing worse economic outcomes. The economic effects of the pandemic—as with the health impacts [6]—vary by race and ethnicity, with Asian, Black, and Hispanic women facing substantively higher rates of continued unemployment compared to White women, at 9.5%, 9.3%, 8.8%, and 5.0%, respectively, as of January 2021 [2]. Thus, the economic recovery for women of color, as well as for women of all backgrounds in harder hit industries, may take substantially longer than it will for more advantaged women [7].

In terms of mental health, Americans in general saw increases in anxiety and depression during the pandemic [8], although effects may have been worse for women than men [9]. Moreover, research suggests that increases in anxiety and worry appear to have been greater among women with children in the household than for men in such households [10-12]. Cameron et al [10] note that risk for maternal anxiety has been particularly vulnerable to financial strain.

Alcohol consumption is known to rise during crises such as pandemic illness. For instance, during the week of March 21, 2020, Nielsen [13] reported that alcohol sales were up 55%. Additional studies have found gender-based differences in alcohol use during the pandemic. Though the prevalence of drinking alcohol, including binge drinking, is generally higher among men than women [14,15], more women than men reported an increased consumption of alcohol since the pandemic began [16]. In fact, the level of pandemic-related distress has shown a positive association with the number of drinks consumed by females in both typical and heavier drinking episodes (16% and 13%, respectively) [17]. Pollard et al [18] found a greater increase in heavy drinking in particular for women compared to men. This increase may be explained by findings that women use alcohol to moderate stress and anxiety more so than do men [19].

In a 2020 survey addressing changes since the onset of the pandemic, 27% of parents reported the emergence of mental health problems and 24% a loss of childcare from March to June. Although this pattern was found evenly across racial, ethnic, income, and education groups, women consistently reported worse perceptions of their own mental health than men [20]. Additionally, since the pandemic began, both men and women reported heavier drinking during the pandemic if children were sheltering at home. This stands in stark contrast to evidence that prepandemic drinking patterns were actually less risky among parents with children at home than those adults without children [21]. This increase in alcohol consumption may be related to the intensive demands of home schooling and daily childcare responsibilities, in addition to the financial and psychological stress already exerted by COVID-19–related lockdowns [12,22-24]. Taken together, relevant studies suggest that the tendency of parents to drink alcohol during COVID-19 is likely to be moderated by pandemic related stress combined with the ongoing demands of childcare and home-based education, which are reportedly more burdensome for females than males.

Cameron et al [10] note in the context of the ongoing pandemic and social distancing directives, internet-based mental health services provide a viable option for families experiencing distress that can afford to access such services. Yet, as the authors report, the transition to remote, telehealth-based psychological interventions has been slow, and moreover, “most telehealth models do not concurrently treat mental health concerns and parenting risks, despite the evidence for the importance of addressing both” [10]. It is in this context, as well as the fact that women are more likely to seek social support online [25], that we have undertaken an examination of alcohol-related content posted by mothers on Instagram. Instagram boasts over 1 billion users per month, with the majority being female [26]. Previous studies have found that posts featuring alcohol consumption are commonplace on social media; however, these studies have tended to focus on posts created by young people, rather than adults in general or mothers specifically [27-29]. At the time of writing, we did not identify any papers in the peer-reviewed literature that examined alcohol-related content posted by mothers on Instagram during the pandemic. Addressing this gap was the purpose of this study, with the aim to better understand the elements of posts with the #winemom and #winejuice hashtags, and to be able to characterize the overall tone and elements of use of #winemom using systematic methods.

## Methods

The methods for this study were similar to others on other health topics [30,31] in that the content on important and timely public health issues was assessed to determine any possible themes present in the data. This study took place in February 2021. Using two popular hashtags, #momjuice and #winemom, 50 Instagram posts on each were collected from the “top posts” tab. At the time of data collection, #momjuice had 37,800 posts and #winemom had 77,600 posts. Posts were excluded if they were in a language other than English (n=3), or if they were advertisements or giveaways (n=9). The date, number of comments, number of likes, presence of a disclaimer (ie, a statement limiting responsibility for the post), and use of an illustration were recorded. The unit of analysis considered images and corresponding captions. Using content analysis, a Microsoft Excel spreadsheet (Microsoft Corp) was created to manually analyze the presence of given themes. Our methods were best defined as follows, “a research technique for the objective, systematic and quantitative description of the manifest content of communication” [32].

The coding categories were created inductively and were as follows: displays alcohol (visible alcohol such as drinking or holding alcohol or alcohol itself), person is making alcoholic beverages (visible ingredients or mixing materials), type of alcohol featured or discussed (if they mentioned or displayed what they were drinking), highlights anxiety and/or depression/mental state (mentions or suggests anxiety, stress, or depression whether in the context of parenting or in general), highlights struggling (mentions or suggests having difficulty overcoming obstacles), highlights parenting challenges (mentions or suggests difficulties specifically related to parenting), encourages alcohol consumption (condones alcohol as beneficial), discourages alcohol consumption (presents alcohol as an unfavorable activity), features a person wearing clothing or shows products promoting alcohol (products ranged from clothing to cups with sayings or words endorsing alcohol consumption), promotes alcohol rehabilitation (mentions or suggests that alcohol rehabilitation is beneficial), highlights caffeine to alcohol daily transition throughout the day (mentions or suggests the need for caffeine early in the day and alcohol later), and highlights other drugs besides caffeine and alcohol (mentions or suggests the use of any other drug).

Interrater reliability was established with a random sample of 10% (or 10 posts) coded by author NQ and recoded independently by author CB. NQ viewed all 100 posts and examined them for a collection of predetermined content characteristics. CB coded a random sample of 10 posts to assess them for the same content. In total, the two reviewers differed in only 4 out of 340 data points. This resulted in near-perfect agreement: an interrater reliability score of =0.96. The 4 discrepancies occurred in the following 3 categories: picture of a child (n=2), highlights struggle (n=1), and wearing clothing or showing products promoting alcohol (n=1). These few discrepancies were resolved through reanalysis of the posts. Data analysis was completed using Microsoft Excel (Microsoft Corp) and included running descriptive statistics and conducting independent one-tailed *t* tests (=.05) on observations of note to determine statistical significance. As this study did not involve human subjects, it did not require approval from the Institutional Review Board at William Paterson University.

## Results

Overall, the 100 reviewed posts had a total of 5108 comments and 94,671 likes. The respective averages were 51.08 (SD 77.94) and 946.71 (SD 1731.72).

Table 1 shows 12 different content characteristics and the total number of posts for which these characteristics were observed. Table 1 also includes the number of comments and likes received by posts featuring this content. Relative percentages are included for comparison.

**Table 1:** Observed content characteristics, comments, and likes of 100 alcohol-related content posted by mothers on Instagram.

Characteristic	Posts (N=100), n	Comments (N=5108), n (%)	Likes (N=94,671), n (%)
Encourages alcohol consumption	66	2762 (54.07)	40,137 (42.40)
Displays alcohol	56	2436 (47.69)	25,779 (27.23)
Highlights struggling	37	1998 (39.12)	50,034 (52.85)
Highlights parenting challenges	26	1394 (27.29)	38,546 (40.72)
Includes clothing or products promoting alcohol	19	1199 (23.47)	5641 (5.96)
Highlights anxiety, depression, or mental state	15	956 (18.72)	20,689 (21.85)
Features a picture of a child	11	419 (8.20)	5428 (5.73)
Discourages alcohol consumption	6	360 (7.05)	3796 (4.01)
Provides a disclaimer	4	700 (13.70)	971 (1.03)
Highlights caffeine to alcohol daily transition	3	120 (2.35)	1856 (1.96)
Features a person making alcoholic beverages	2	97 (1.90)	1136 (1.20)
Highlights other drugs besides caffeine and alcohol	2	395 (7.73)	11,133 (11.76)

A majority (>50%) of the posts we reviewed encouraged alcohol consumption (n=66) and/or displayed alcohol (n=56). Of the 66 that encouraged and/or displayed alcohol, the common type of alcohol discussed or featured was wine (n=55). Only 6 posts reviewed discouraged alcohol use, and only 4 provided the audience with a disclaimer. None of the posts promoted or endorsed alcohol rehabilitation in any way. Therefore, this characteristic was removed from the table.

Even though more than 50% of the posts reviewed displayed alcohol, these posts only garnered 26.95% (n=25,779) of the total likes. An independent one-tailed *t* test (=.05) showed this observation to be statistically significant (*P*=.002). More specifically, the *t* test showed that posts that displayed alcohol were less likely to receive a like when compared to those posts that did not display alcohol. The average number of likes for posts displaying alcohol was 460.34 (SD 1006.56) compared to 1565.73 (SD 2231.14) for posts not displaying alcohol.

Only 37% of the posts reviewed highlighted struggle. However, these posts garnered more than a majority of the likes (n=50,034, 52.3%). Posts that showed struggle received an average of 1359.57 (SD 2108.02) likes. Those that did not show struggle had an average of 704.24 (SD 1447.46) likes. An independent one-tailed *t* test (=.05) showed this difference to be statistically significant (*P*=.0499) as well. Therefore, the data indicate that posts highlighting struggle were more likely to receive likes than those that do not show struggle. It should be noted that the World Health Organization declared that COVID-19 had reached pandemic levels on March 11, 2020 [33]. A total of 23 posts occurred before the pandemic declaration (prior to March 11), and 77 posts occurred afterwards (on and after March 11). Of the 23 posted before the pandemic declaration, 19 (82.61%) did not have a theme of struggling and 4 (21.05%) did. Of the 77 posted during the pandemic, 44 posts (57.14%) did not highlight struggling, whereas 33 (42.86%) did. Of the 37 posts that highlighted struggle, 13 (35.14%) also displayed alcohol. None of these 13 posts displayed a person making an alcoholic beverage. However, 11 of these posts (84.61%) did encourage the consumption of alcohol.

## Discussion

Our findings suggest that the sample of posts evaluated in this study, under the hashtags #momjuice and #winemom, most commonly indicated encouragement of alcohol consumption and display of alcohol, and highlighted coping struggles. The fact that content related to struggling garnered more likes than posts encouraging alcohol use suggests that #winemom and #momjuice may provide a forum for validation and support related to the burdens faced by mothers trying balance multiple forms of labor—paid and unpaid. Notably, while some of the posts in our sample were dated prior to the declaration that COVID-19 as a pandemic, those that occurred after were more likely to highlight struggling. This may be indicative of the additional “care economy” work [5] required by mothers over the past year.

While it is important to note that the “wine mom” terminology existed prior to the pandemic [34], the proliferation of “wine mom” and “mom juice” paraphernalia [35] leads to questions as to the reasons behind the movement. It is currently unknown whether the derivation of the #winemom and #momjuice movement is simply a humorous meme with limited implications, or if there is more to the message that should take into account the undue pressure placed on all parents, particularly mothers, during the COVID-19 pandemic [34-37]. In times of crisis, individuals who participate socially (eg, identify with groups, derive social support from others, feel a sense of belonging to a community) may benefit from enhanced personal resources [38,39]. Online groups such as “wine mom” may thus function as a humorous protective buffer for its members. Along with the social connection provided by the group, the humorous aspect, as well as the situational reframing, may provide a relieving counterpoint to the strong negative emotions felt by many as the pandemic unfolded, lockdowns were mandated, and women in particular faced sudden and dramatic changes in roles and perceptions of mental health [40,41].

This study is limited by the small sample size, the cross-sectional design, and the ever-evolving state of posts on this platform. Further study should focus on commentary generated on these posts as well as how these may change on a longitudinal basis. As with all cross-sectional studies, external validity is low. Further, our methodology was limited by the lack of profile data on the source of each post. Nevertheless, to our knowledge, this is the first study to examine this content in general, and specifically during a time of heightened stress and anxiety. The findings of this investigation suggest that though these hashtags ostensibly exist to valorize excess alcohol consumption, they may be serving as a support system for mothers who are experiencing increased burdens and role stress during the pandemic. Given the strains placed on mothers overall and especially during the COVID-19 pandemic, efforts must be taken to increase access to and affordability of telehealth-based mental health care. Social media forums such as Instagram are a place to potentially highlight the availability of such services.
